# Supplementary material for: A Reproducible Protocol to Assess Arrhythmia Vulnerability in silico: Pacing at the End of the Effective Refractory Period
Source: Front Physiol. 2021 Apr 1;12:656411. doi: 10.3389/fphys.2021.656411 (PMC8047415; doi:10.3389/fphys.2021.656411)
Supplement: Supplementary file 1 [file Data_Sheet_1.PDF]

Supplementary Material for
A Reproducible Protocol to Assess Arrhythmia Vulnerability in silico: Pacing at the End of the Effective Refractory Period

Luca Azzolin*, Steffen Schuler, Olaf Dössel, Axel Loewe

Institute of Biomedical Engineering, Karlsruhe Institute of Technology (KIT)

Correspondence: publications@ibt.kit.edu

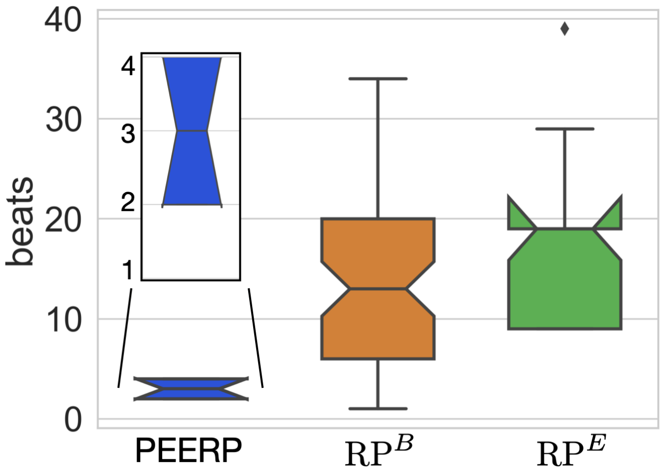

Figure S1. Total number of beats applied in the protocols PEERP, RP^B and RP^E inducing arrhythmic episodes in the model H4B.

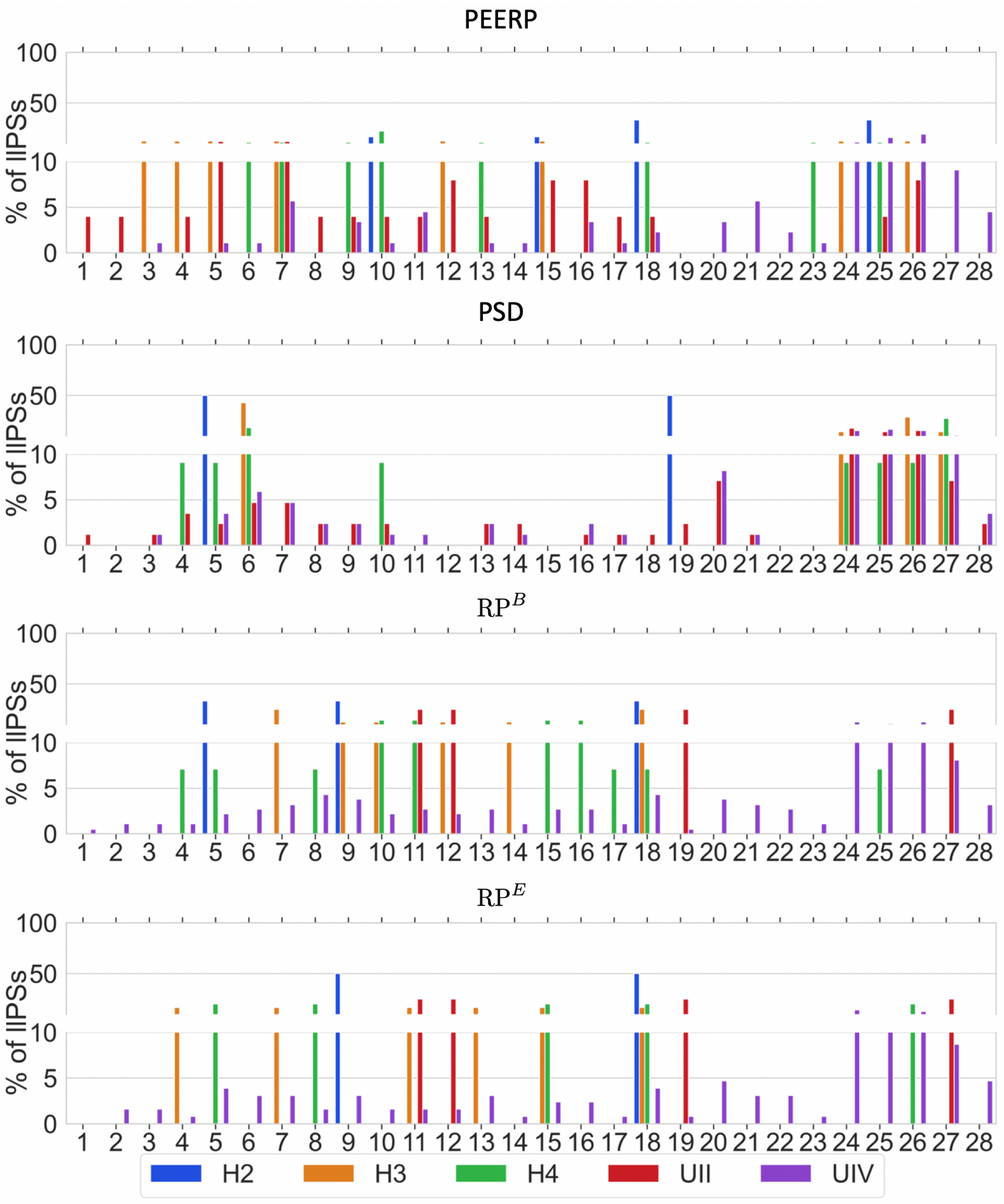

Figure S2. Atrial segments in which inducing points were identified applying the various protocols in the different models.

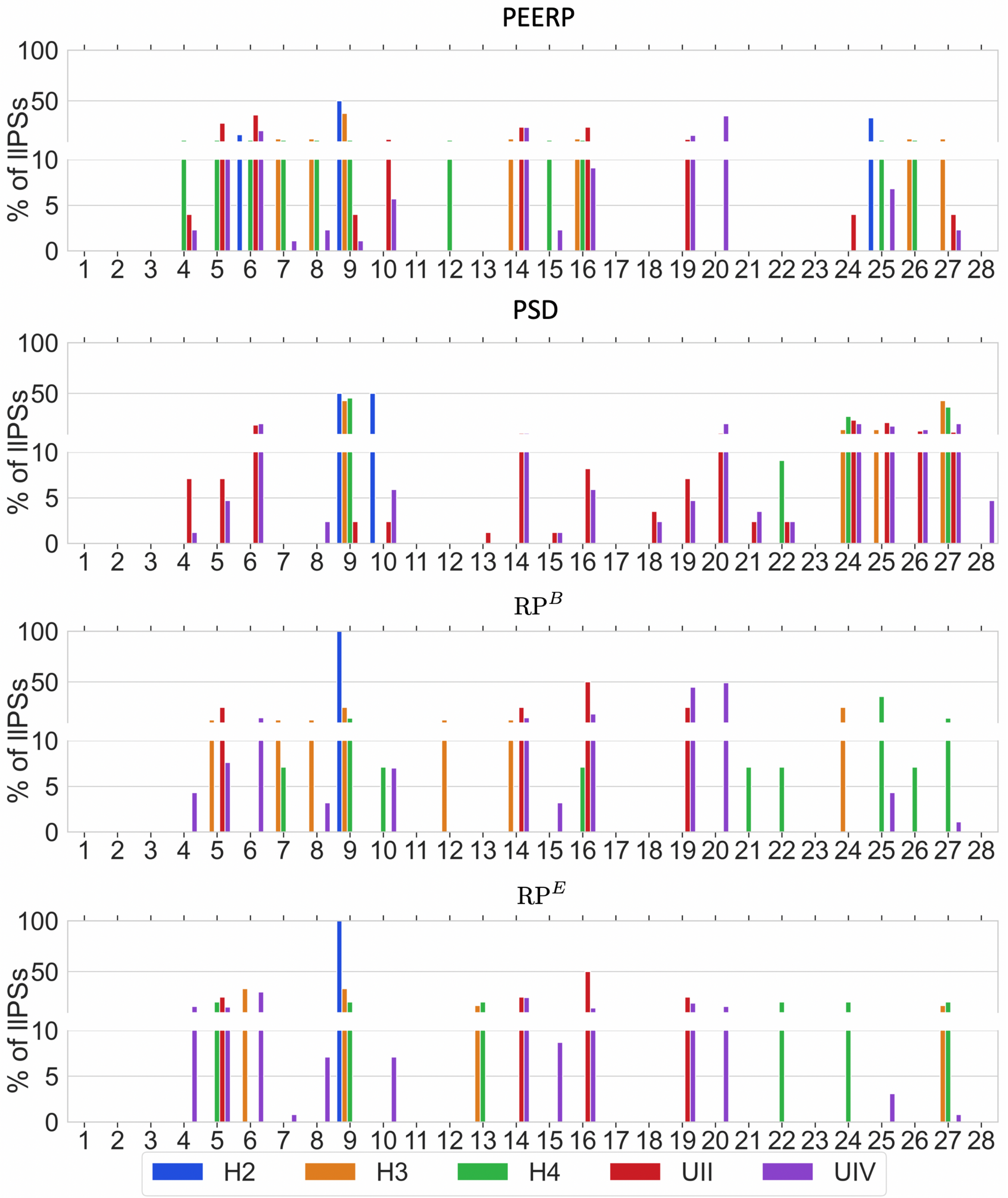

Figure S3. Atrial segments in which stable llPSs were maintained in the different models applying each protocol.
